# Postoperative cellular stress in the kidney is associated with an early systemic γδ T-cell immune cell response

**DOI:** 10.1186/s13054-018-2094-x

**Published:** 2018-07-04

**Authors:** Ivan Göcze, Katharina Ehehalt, Florian Zeman, Paloma Riquelme, Karin Pfister, Bernhard M. Graf, Thomas Bein, Edward K. Geissler, Piotr Kasprzak, Hans J. Schlitt, John A. Kellum, James A. Hutchinson, Elke Eggenhofer, Philipp Renner

**Affiliations:** 1 Department of Surgery, Surgical Intensive Care and Experimental Surgery, University Medical Center Regensburg, Regensburg, Germany; 2 Department of Anesthesia, University Medical Center Regensburg, Regensburg, Germany; 3 Center for Clinical Studies, University Medical Center Regensburg, Regensburg, Germany; 4 Department of Vascular and Endovascular Surgery, University Medical Center Regensburg, Regensburg, Germany; 5 Center for Critical Care Nephrology, Department of Critical Care Medicine, University of Pittsburgh, Pittsburgh, PA 15621 USA

**Keywords:** Tubular cell stress, Aortic surgery, Immunosurveillance, γδ T cells, Ischemia-reperfusion injury

## Abstract

**Background:**

Basic science data suggest that acute kidney injury (AKI) induced by ischemia-reperfusion injury (IRI) is an inflammatory process involving the adaptive immune response. Little is known about the T-cell contribution in the very early phase, so we investigated if tubular cellular stress expressed by elevated cell cycle biomarkers is associated with early changes in circulating T-cell subsets, applying a bedside-to-bench approach.

**Methods:**

Our observational pilot study included 20 consecutive patients undergoing endovascular aortic repair for aortic aneurysms affecting the renal arteries, thereby requiring brief kidney hypoperfusion and reperfusion. Clinical-grade flow cytometry-based immune monitoring of peripheral immune cell populations was conducted perioperatively and linked to tubular cell stress biomarkers ([TIMP-2]•[IGFBP7]) immediately after surgery. To confirm clinical results and prove T-cell infiltration in the kidney, we simulated tubular cellular injury in an established mouse model of mild renal IRI.

**Results:**

A significant correlation between tubular cell injury and a peripheral decline of γδ T cells, but no other T-cell subpopulation, was discovered within the first 24 hours (*r* = 0.53; *p* = 0.022). Turning to a mouse model of kidney warm IRI, a similar decrease in circulating γδ T cells was found and concomitantly was associated with a 6.65-fold increase in γδ T cells (*p* = 0.002) in the kidney tissue without alterations in other T-cell subsets, consistent with our human data. In search of a mechanistic driver of IRI, we found that the damage-associated molecule high-mobility group box 1 protein *HMGB1* was significantly elevated in the peripheral blood of clinical study subjects after tubular cell injury (*p* = 0.019). Correspondingly, *HMGB1* RNA content was significantly elevated in the murine kidney.

**Conclusions:**

Our investigation supports a hypothesis that γδ T cells are important in the very early phase of human AKI and should be considered when designing clinical trials aimed at preventing kidney damage.

**Trial registration:**

ClinicalTrials.gov, NCT01915446. Registered on 5 Aug 2013.

**Electronic supplementary material:**

The online version of this article (10.1186/s13054-018-2094-x) contains supplementary material, which is available to authorized users.

## Background

Discovery of the cell cycle arrest biomarkers TIMP-2 and IGFBP7 (tissue inhibitor of metalloproteinase 2 and insulin-like growth factor-binding protein 7, respectively) enables the early detection of tubular cellular stress [1]. When exposed to stress, epithelial tubular cells may enter a short period of G_1_ cell-cycle arrest until the danger has passed or the injury has been repaired. The urinary biomarkers TIMP-2 and IGFBP7, which are released in stressed cells as an “alarm signal” for adjacent cells, have recently been established as early predictors of imminent acute kidney injury (AKI) in general and postoperative patients [2, 3]. The initiation of early renal protection strategies in patients with elevated stress markers TIMP-2 and IGFBP7 has recently reduced the incidence and severity of postoperative AKI, thus improving the short-term outcome in interventional randomized trials [4, 5].

Stressed tubular cells may be sensed by the immune system, similar to oncogenic stress in cancer cells. In cancer, to prevent malignant cellular transformation, stressed human cells are protected through cell-intrinsic barriers, such as induction of cell-cycle arrest and apoptosis, and cell-extrinsic barriers, such as recognition by the immune system and immune elimination [6]. In AKI that has been recognized as a proinflammatory state, equivalent scenarios may exist. Describing this early immune response pathway could have an important impact on future research, including the development of novel therapeutic interventions. Current data suggest that T cells play a key role in the initiation and propagation of the immune reaction in experimental AKI [7, 8].

Therefore, we investigated in our pilot study if tubular cellular stress expressed by elevated cell-cycle arrest biomarkers is associated with early changes in circulating T-cell subsets, using a bedside-to-bench approach. The aim of the study was to obtain the first evidence that this association exists. Renal ischemia-reperfusion injury (IRI) is significantly associated with AKI and also occurs during various surgical procedures in humans, including the treatment of suprarenal aortic aneurysm. In the first part of our study, we evaluated changes in circulating T-cell subsets and their association with tubular cell stress as measured by [TIMP-2]•[IGFPB7] in a clinical setting. Subsequently, findings from the clinical setting were further validated in murine kidneys within the first 24 hours after renal IRI in the experimental part of the study. This bedside-to-bench approach was used to prove the presence of immune cells in kidney tissue and to confirm findings of the pilot clinical study.

## Methods

The study was performed between November 2013 and May 2017 at the University Hospital Regensburg.

### Clinical study and patients

The clinical part of the study was performed first. In this observational study, we included 20 consecutive patients undergoing multibranched endovascular thoracoabdominal aortic repair. Occlusion and intraluminal manipulation of the renal artery during multibranched endovascular aortic repair lead to a brief episode of kidney hypoperfusion and reperfusion. Two patients were excluded: one because of intraoperative interruption of aneurysm repair owing to technical difficulties and one because of requirement of urgent reoperation < 24 hours after surgery. Eligible patients fulfilled the following four criteria: (1) adults (aged 18 years or older), (2) elective repair of thoracoabdominal aortic aneurysm, (3) presence of a central venous catheter for repeated blood collection, and (4) provision of written informed consent. Exclusion criteria were (1) preexisting liver and kidney failure, defined as a spontaneous international normalized ratio > 2 and encephalopathy, or serum creatinine (SCr) > 2 mg/dl, or preoperative requirement of renal replacement therapy; and (2) anemia with hemoglobin concentration of < 7 g/dl.

Ultrasound imaging was conducted at the bedside in a standardized manner. A conventional ultrasound B-scan was followed by color-coded Doppler sonography. Renal perfusion was quantified by mean values of renal resistive index measured in the renal artery and in the interlobar arteries. All examinations were supervised and analyzed at the bedside by a board-certified sonographist, according to German Society for Ultrasound in Medicine guidelines. Postoperative AKI was defined according to the 2012 Kidney Disease: Improving Global Outcomes criteria [9].

The primary endpoint was change in circulating T-cell subsets in subjects with tubular cellular stress. The key secondary endpoints were incidence of AKI, changes in circulating T cells and cellular infiltration of murine kidneys in histologically proven tubular injury, and values of high-mobility group box 1 (*HMGB1*) in the clinical and experimental studies.

### Collection and analysis of blood and urine

Blood samples for measuring laboratory parameters such as SCr, creatinine kinase, lactate, hemoglobin, interleukin (IL)-6, C-reactive protein, and central venous oxygen saturation were collected by central venous sampling after the induction of anesthesia, before skin incision, and at prespecified points after surgery (less than 4 hours after the end of surgery and 24 hours after the start of surgery). Urinary [TIMP-2]•[IGFBP7] was measured simultaneously after surgery by means of the immunoassay method integrated in the Astute 140 Meter Kit (Nephrocheck test; Astute Medical Inc., San Diego, CA, USA). A cutoff value of [TIMP-2]•[IGFBP7] ≥ 0.3 was used to identify tubular cellular stress and to predict AKI [10]. All values for [TIMP-2]•[IGFBP7] are reported in units of (ng/ml)^2^/1000. Blood samples for immune monitoring were collected directly into a Cyto-Chex® BCT tube (Streck, Omaha, NE, USA) by central venous sampling after the induction of anesthesia, before skin incision, and 24 hours after the start of surgery. Immediately after collection, the contents of the Cyto-Chex® tube were mixed by gentle inversion, and the tube was immediately transported to the in-house immune monitoring laboratory. Samples were typically processed within 30 minutes after blood collection; however, standard operating procedures allowed samples to be stored at 4 °C for up to 4 hours before processing. To prepare samples for analysis, 100 μl of whole blood was transferred from the Cyto-Chex® tube into a conical tube (Sarstedt, Nürnbrecht, Germany). Unspecific binding was blocked with 10 μl of human FcR blocking reagent (Miltenyi Biotec, Bergisch Gladbach, Germany) in DPBS without Ca^2+^ or Mg^2+^ (PAA Laboratories, Cölbe, Germany) and incubated on ice for 30 minutes. Primary conjugated antibodies were added in optimized amounts to stain the cells at 4 °C for 1 hour. To remove erythrocytes, samples were incubated in 2 ml of 1 × BD lysing solution (BD Biosciences, Heidelberg, Germany) at 4 °C for 10 minutes before being washed twice with 2 ml of DPBS. Samples were analyzed using a FACSCanto II cytometer (BD Biosciences) within 30 minutes of antibody staining. As an internal stability control for cytometer performance, a BD™ Cytometer Setup and Tracking (CST) bead assay (BD Biosciences) was conducted before each analysis. As many CD45^+^ events as possible were recorded. Data were analyzed by a blinded operator using FlowJo® software (FlowJo, Ashland, OR, USA).

### Murine ischemia-reperfusion model

The experimental study was performed after analysis of the clinical data. Six- to 8-week-old C57BL/6 mice (Charles River Laboratories, Sulzfeld, Germany) underwent kidney IRI as described elsewhere [11]. In brief, the renal vessels were exposed bilaterally through a midline incision and occluded with vascular clamps for 30 minutes. During the procedure, animals were kept at a constant temperature (37 °C) and hydrated with warm saline. After 24 hours, the mice were killed, and their organs were harvested for further analysis. Sham-operated animals (controls) were treated identically except for renal artery occlusion. All animal experiments were conducted upon approval by the Government of Upper Palatinate.

### Flow cytometry

In mice, spleens and kidneys were harvested and processed to single-cell suspensions. Renal leukocytes were isolated by mechanically disrupting renal tissue in 5 ml of Paris buffer (20 mM Tris-HCl,125 mM NaCl, 10 mM KCl, 10 mM glucose, pH 7.4). To remove debris, samples were passed through a 100-μm cell strainer (Falcon). The resulting cell suspension was centrifuged at 300 × *g* for 10 minutes to pellet the cells. The pellet was then suspended in 36% Percoll (Amersham Pharmacia, Stockholm, Sweden), gently overlaid onto 72% Percoll and centrifuged at 1000 × *g* for 30 minutes at room temperature. Cells were isolated from the Percoll interface and washed twice in magnetic-activated cell sorting (MACS) buffer at 300 × *g* at 4 °C for 10 minutes. Samples were resuspended in 250 μl of MACS buffer. The number of leukocytes was assessed using trypan blue exclusion on a hemocytometer. Single-cell suspensions were then stained with antibodies against CD3, CD4, CD8, and γδ T-cell receptor (eBiosciences, Frankfurt, Germany), and antigen expression was determined with a FACSCanto flow cytometer (BD Biosciences). Data were analyzed with FlowJo software.

### Enzyme-linked immunosorbent assay

Human *HMBG1* serum levels were analyzed with a commercially available *HMGB1* enzyme-linked immunosorbent assay kit (IBL International, Hamburg, Germany) according to the manufacturer’s instructions.

### Real-time PCR

Murine kidney pieces were collected in RNA*later* buffer (Qiagen, Hilden, Germany) for further analysis. Complementary DNA (cDNA) was isolated from murine kidney samples using the μMACS ONEstep cDNA Kit (Miltenyi Biotec). Relative gene expression changes between *GAPDH* and *HMGB1* were determined by means of the comparative cycle threshold method. The following QuantiTect Primer Sets (Qiagen) were used: QT01658692 for *GAPDH* and QT02520210 for *HMGB1*.

### Statistical analysis

No formal sample size calculation was done for the pilot study. A sample size of 20 was expected to yield sufficient power to detect first effects and correlations. Categorical data are presented as absolute numbers and percentages. Continuous data are summarized as mean (SD) or as median (IQR) and were compared using the nonparametric Mann-Whitney *U* test, if not stated otherwise. Pearson’s correlation coefficient was used to assess correlations between continuous variables. The level of significance was set to 5% without any adjustments for multiple testing. Analyses were conducted using Prism version 6 software (GraphPad Software, La Jolla, CA, USA) and R version 3.2.1 (R Foundation for Statistical Computing, Vienna, Austria).

## Results

### In patients, tubular cellular stress/injury was associated with loss of function

Baseline characteristics are shown in Table 1. A schematic overview of our diagnostic trial is given in Additional file 1: Figure S1a. Nine (50%) of 18 patients undergoing endovascular aortic repair showed elevated urinary cell stress biomarkers [TIMP-2]•[IGFBP-7] within 4 hours after surgery. On the day after surgery, patients with elevated biomarkers showed a 1.53-fold (SD 0.33) mean increase in creatinine levels compared with baseline values, whereas patients with physiological biomarker levels only showed a 1.13-fold (SD 0.11) mean elevation (Additional file 1: Figure S1b) (*p* = 0.021) and thus had a significantly higher rate of AKI (OR, 12.25; 95% CI, 1.33–113.06; *p* = 0.027).

**Table 1:** Baseline perioperative characteristics and outcome parameters

Variables	[TIMP-2•[IGFBP7] < 0.3 (*n* = 9)	[TIMP-2]•[IGFBP7] ≥ 0.3 (*n* = 9)	*P* value
Age, yr, median (IQR)	66.7 (58.3–71.6)	73.4 (69.9–73.9)	0.070
BMI, kg/m^2^, median (IQR)	28.4 (24.8–31.0)	28.1 (24.8–31.6)	1.00
Sex, female	2	3	0.624
Diabetes mellitus	1	2	0.527
Smoking	6	5	0.629
Coronary heart disease	4	5	0.637
Hypertension	8	8	1.000
SAPS II, median (IQR)	27 (20.5–40.0)	34 (27.0–53.5)	0.112
SOFA score at admission, median (IQR)	4.0 (1.0–8.0)	8.0 (5.0–9.5)	**0.045**
Ventilation days	0.0 (0.0–1.05)	0.75 (0.0–14.7)	0.219
Preoperative IL-6, mg/dl, median (IQR)	6.15 (5.22–20.95)	6.8 (5.55–13.10)	0.847
Preoperative hemoglobin, g/dl, median (IQR)	12.8 (11.15–14.25)	12.9 (11.05–13.75)	0.965
Preoperative lactate, mg/dl, median (IQR)	7.4 (6.00–8.50)	8.0 (7.00–8.60)	0.473
Preoperative ScvO_2_, %, median (IQR)	82.0 (77.00–86.65)	86.4 (84.35–87.85)	0.077
Intraoperative values, median (IQR)			
Duration of surgery, min	281.0 (203.5–347.0)	422.0 (303.0–482.5)	**0.031**
Intravenous contrast agent, ml	240.0 (150.0–262.5)	250.0 (179.25–337.0)	0.469
Urine output, ml/h/kg	1.6 (1.50–2.80)	2.9 (1.70–4.75)	0.132
Fluid balance, ml	2420 (1850–3125)	3350 (2915–4474)	**0.022**
Blood transfusion, ml	0.00 (0.00–0.00)	600.0 (0.00–1500)	**0.014**
Noradrenalin, mg	0.85 (0.34–2.2)	4.6 (1.65–6.67)	**0.012**
MAP below 65 mmHg, %	0.00 (0.00–7.00)	0.00 (0.00–1.90)	0.599
Postoperative values, median (IQR), at admission ICU			
Noradrenalin μg/kg/min	0.06 (0.0–0.15)	0.23 (0.07–0.30)	**0.046**
Creatinine, mg/dl	1.11 (0.95–1.39)	0.87 (0.71–1.09)	0.112
IL-6, mg/dl	66.0 (43.25–276.45)	406.9 (84.08–856.25)	0.102
Hemoglobin, g/dl	11.0 (9.3–13.35)	10.2 (9.80–12.35)	0.627
Lactate mg/dl	9.8 (6.10–13.95)	15.0 (9.5–18.60)	0.170
ScvO_2_, %,	76.2 (71.35–79.25)	78.4 (68.3–81.80)	0.427
Postoperative values, median (IQR), at 24 h			
Mean arterial pressure 0–24 h after admission	76.1 (72.4–87.7)	75.7 (68.5–84.7)	0.310
Doppler RI both kidneys at 24 h	0.73 (0.67–0.76)	0.73 (0.72–0.80)	0.232
IL-6, mg/dl, at 24 h	167.8 (31.75–221.00)	185.1 (36.20–633.25)	0.354
Hemoglobin, g/dl, at 24 h	10.1 (8.35–12.5)	9.6 (8.4–11.05)	0.930
Lactate, mg/dl, at 24 h	11.4 (6.55–14.95)	14.0 (10.00–31.00)	0.122
ScvO_2_, %, at 24 h	68.9 (65.4–74.65)	76.2 (68.05–81.25)	0.200
Furosemide dose 0–24 h, mg	10.0 (0.0. 25.0)	30.0 (5.0–45.0)	0.133
Outcome parameters			
AKI	2	7	**0.02**
AKI Stage 2 and 3	0	3	0.058
RRT	0	2	0.134
In-hospital mortality	0	2	0.134

*Abbreviations: AKI* Acute kidney injury, *BMI* Body mass index, *CHD* Coronary heart disease, *CKD* Chronic kidney disease, *CK* Creatinine kinase, *CRP* C-reactive protein, *Hb* Hemoglobin, *IL-6* Interleukin 6, *MAP* Mean arterial pressure, *RRT* Renal replacement therapy, *SAPS* Simplified Acute Physiology Score, *SCr* Serum creatinine, *ScvO*_*2*_ Central venous oxygen saturation, *TIMP-2* Tissue inhibitor of metalloproteinase, *IGFBP7* Insulin-like factor-binding protein, *RI* Resistive index

Data are for patients with [TIMP-2]•[IGFBP7] low versus high

Note: Bold *p* values represent a statistically significant difference between the two groups

Postoperative renal perfusion was similar in both biomarker groups, as shown by mean arterial pressure over the first 24 hours and standard Doppler sonography. Patients who developed early tubular cell stress required longer duration of surgery and more blood transfusions and had a more positive intraoperative fluid balance, higher vasopressor dose, and higher SOFA score at admission than patients with urinary [TIMP-2][IGFBP-7] < 0.3 (Table 1, Additional file 1: Figure S1c).

### γδ T cells were decreased in patient blood with elevated biomarkers

**Fig. 1:**
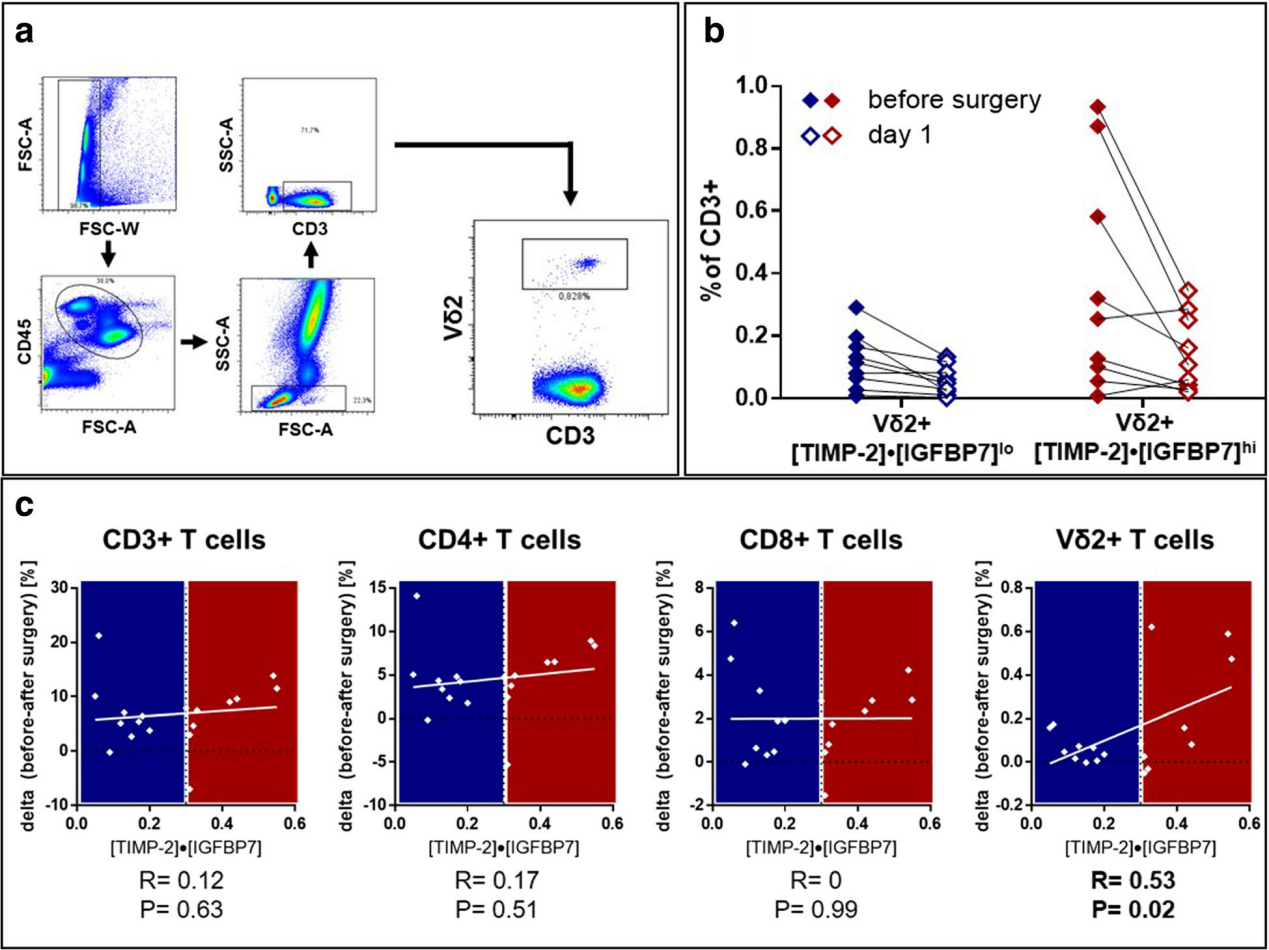
Correlation between human γδ T-cell subsets and biomarkers of kidney stress. **a** Gating strategy used for flow cytometric analysis. Following exclusion of doublets, CD45^+^ cells were analyzed and further specified according to size/granularity. γδ T cells were defined as CD3^+^Vδ2^+^ cells. **b** Perioperative changes in human peripheral γδ T-cell frequencies were analyzed and stratified by biomarker results. In patients with elevated biomarkers, greater changes could be observed. **c** Perioperative changes (postsurgery versus presurgery) of T-cell subsets in human peripheral blood, including γδ T cells, were correlated with the biomarker level ([TIMP-2]•[IGFBP7]). Here, only changes in the γδ T-cell subpopulation significantly correlated with biomarker testing (*p* = 0.022), whereas no significant correlation could be observed in the (global) CD3 population or CD4/CD8 T-cell subsets

All patients underwent clinical-grade standardized immune monitoring (Fig. 1a). When comparing γδ T-cell frequencies before and after surgery, we found that declines in γδ T-cell frequencies were more pronounced in the patient subset with elevated renal biomarkers (Fig. 1b). Strikingly, the decrease in γδ T cells from the circulation correlated with the value of urinary biomarkers (Fig. 1c) (*r* = 0.53. *p* = 0.022). There was no correlation of postoperative changes with other T-cell subsets in the first 24 hours. Similar results were found by evaluation of the absolute cell count (Additional file 2: Figure S2) (*r* = 0.60, *p* = 0.01).

### Murine IRI leads to rapid γδ T-cell infiltration of the kidneys

**Fig. 2:**
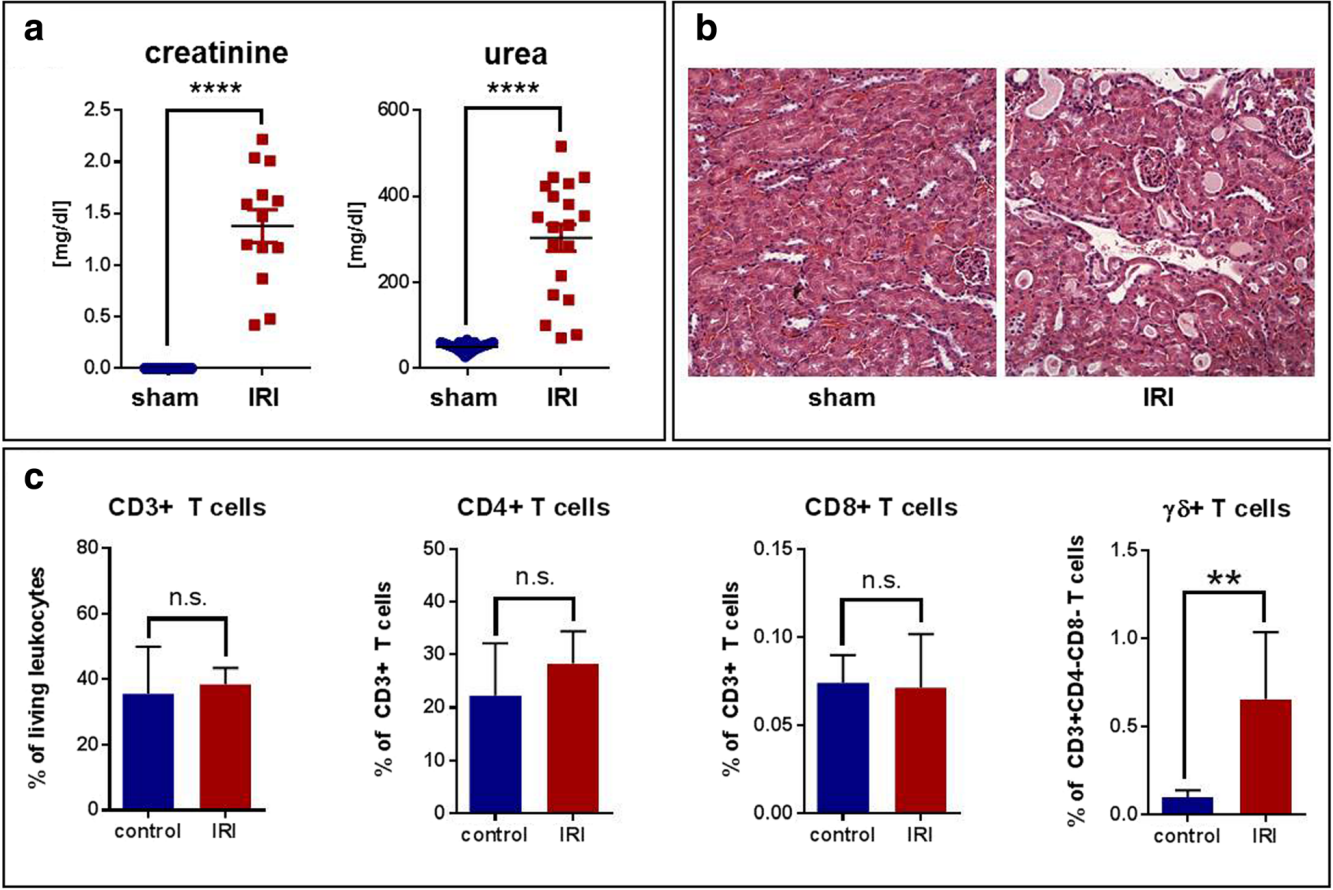
Murine ischemia-reperfusion model. **a** Thirty minutes of warm ischemia induced a highly significant increase in creatinine (left) and urea (right) after 24 hours. **b** Accordingly, histology (H&E staining) showed acute tubular injury with immune cell infiltration. Original magnification 200 ×. **c** Characterization of T-cell subsets isolated from murine kidneys (*n* = 6 per group) showed that γδ T cells, in contrast to other T-cell subpopulations, were significantly increased after ischemia-reperfusion injury. Data are shown as mean values with SEM. The Mann-Whitney *U* test was used to determine statistical significance. *n.s.* Not significant. **p < 0.01; ****p < 0.0001

**Fig. 3:**
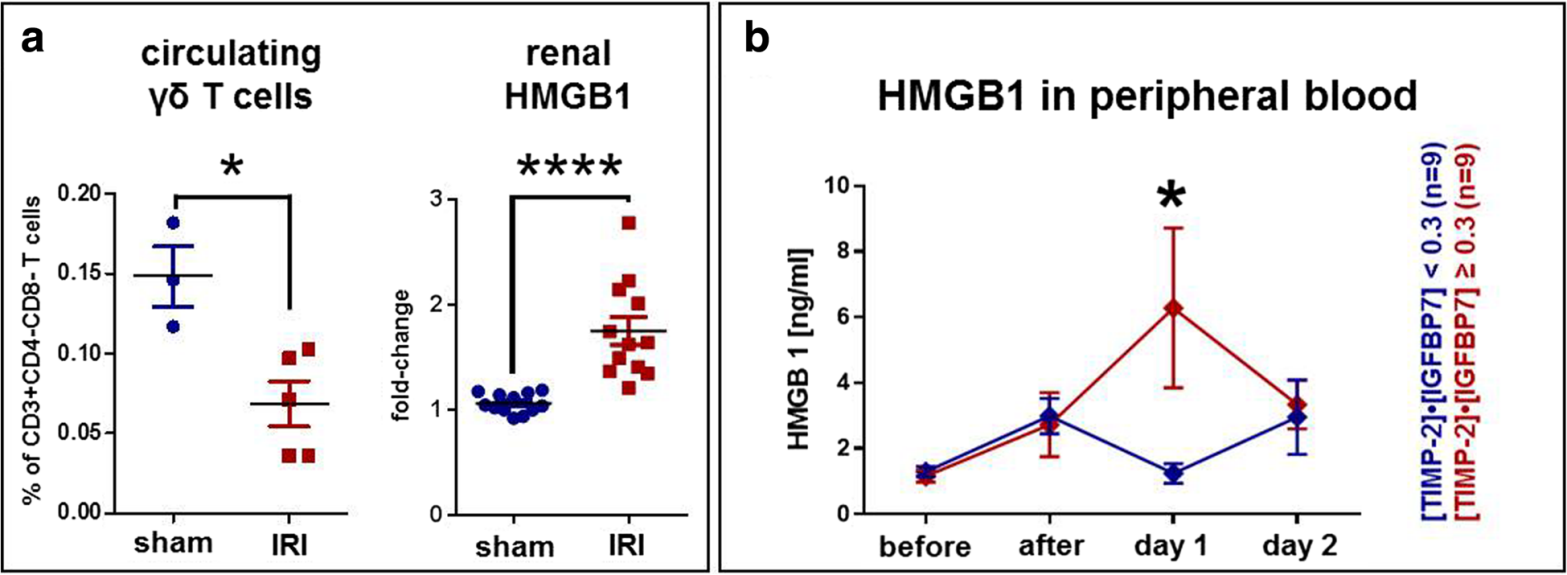
*HMGB1* as a potential driver of γδ T-cell response. **a** In the murine ischemia-reperfusion injury (IRI) model, the pattern of peripheral γδ T-cell changes was similar to that in the human samples (*see* Fig. 1c). Here, peripheral γδ T-cell frequency was significantly reduced after IRI (left, *p* = 0.036). Interestingly, *HMGB1* gene expression was significantly increased in murine kidneys (right, *p* < 0.0001). **b** In study participants, the *HMGB1* protein was increased in the peripheral blood on day 1 after surgery. Strikingly, biomarker-positive study subjects had significantly higher *HMGB1* levels on day 1 than biomarker-negative patients (*p* = 0.019). All data are shown as means with SEM and were evaluated for significance using the Mann-Whitney *U* test

C57BL/6 mice showed a markedly increased level of serum creatinine and urea within the first 24 hours after standardized warm ischemia (Fig. 2a). In parallel, murine kidneys showed histopathological features of early kidney injury (Fig. 2b). Lymphocytes infiltrated the renal interstitium, and infiltrates were analyzed by flow cytometry. γδ T cells were increased 6.65-fold compared with sham-operated controls (*p* = 0.002) in ischemic kidneys within the first 24 hours after injury, whereas levels of other T-cell subsets tested were similar in both groups (Fig. 2c). Similarly to the human setting, peripheral (blood) murine γδ T cells were significantly reduced in IRI mice as compared with controls (Fig. 3a left), showing that γδ T cells home from blood to the kidneys after cellular injury and induction of IRI.

### *HMGB1* correlates with murine IRI and human expression of [TIMP-2]•[IGFBP7]

In search of a mechanistic driver of γδ T-cell homing, we investigated the role of *HMGB1*. *HMGB1* has recently been described as a potent proinflammatory cytokine released from ischemia-reperfusion-injured cells. *HMGB1* messenger RNA (mRNA) expression was significantly increased in murine kidneys within the first 24 hours after IRI (Fig. 3a, right). In humans, *HMGB1* protein was significantly elevated in biomarker-positive patients 1 day after surgery compared with biomarker-negative patients (median [IQR] 0.87 [0.68–1.82] versus 2.63 [1.59–12.20] ng/ml, *p* = 0.019) (Fig. 3b).

## Discussion

In the present study, we investigated the role of human T cells in patients with renal tubular cellular stress and found that the γδ T-cell subset is involved in the first-line immune response in the very early phase of AKI in both humans and mice. We were able to link the decrease in circulating γδ T cells within first 24 hours to elevated cellular stress biomarkers TIMP-2 and IGFBP7 measured 4 hours after surgery, suggesting that stressed/injured tubular renal cells are recognized early by immune stress surveillance. Accordingly, a drop of γδ T cells in the blood of mice was associated with significant cellular infiltration in the murine kidneys within the first 24 hours after cellular injury, suggesting that γδ T cells traffic from the blood compartment to injured kidneys. Finally, *HMGB1* was elevated in mice and humans and may serve as a link between local cellular injury and aggravation of the systemic inflammatory response.

Injured epithelial cells are recognized by lymphoid stress immunosurveillance [12, 13]. Interestingly, in humans, γδ T cells as a minor fraction of T cells in peripheral blood [14–16] are of increasing importance in early first-line defense in various tissues, such as the skin, brain, liver, and lungs [17–20]. The presence of these cells in murine kidneys has been reported previously, but their role in human AKI is unclear [21].

In patients, perioperative decrease in circulating human γδ T-cell subpopulations within the first 24 hours after surgery correlated with the values of cell injury biomarkers in our study. No other T-cell subset showed this early correlation. Because only biopsy specimens prove the presence of γδ T cells in human kidneys during early AKI, and because biopsies are not obtained in the care of these patients, we simulated tubular cell injury in a mouse model of mild renal IRI to prove γδ T-cell infiltration in the kidney. Characterization of T-cells subsets isolated from murine kidneys showed that γδ T cells were significantly increased within the first 24 hours after IRI, suggesting that these cells traffic to injured kidneys [22]. No early expansion of other tested detected T cells was observed. Therefore, γδ T cells appear to act as a mediator of early renal IRI, whereas other T-cell subsets, such as CD4 and CD8 cells, are major likely effectors of the immune response at a later stage [23]. This finding may also suggest that the immune response is triggered immediately after cellular stress/injury, hours before the alteration of kidney function can be detected with standard functional markers (such as serum creatinine). Indeed, in a clinical scenario, these cells can be activated upon aseptic tissue damage because they sense danger signals such as phospholipids or markers of cell injury [24]. A very recent analysis of 39 cancer types has shown that intratumoral γδ T cells are the most significant favorable prognostic immune population owing to their response to stress signals expressed by tumor cells [14]. γδ T-cell-based immunotherapy has been well tolerated and efficient in clinical trials in patients with advanced renal cell carcinoma [25], underscoring the potential therapeutic use of this cell population for other diseases as well.

Although our findings in both the mouse model and the clinical setting showed that γδ T cells are involved in the early immune response to kidney injury, it is not clear how this response is triggered. *HMGB1* protein released from injured cells may either induce cell-cycle arrest in epithelial cells [26] or modulate the immunological cascade through activating pattern recognition receptors in immune cells [22]. Therefore, HMBG1 may serve as a link between cell damage and activation of the systemic inflammatory response [27, 28]. Indeed, *HMGB1* mRNA expression was significantly increased in murine kidneys within 24 hours after IRI in our study. Analogously, surgical patients showed a marked *HMGB1* increase in [TIMP-2]•[IGFBP7] biomarker-positive patients after 1 day. Although our findings may support the hypothesis that *HMGB1* is released from injured epithelial tubular cells, we have not yet examined whether *HMGB1* is the driver of γδ T-cell expansion. However, *HMGB1* has already been shown to be critical in inducing IL-17-producing γδ T cells via Toll-like receptor 4 (TLR4) activation on macrophages and blockade of *HMGB1* suppressed IL-17^+^ γδ T-cell response [20, 29]. Furthermore, damage-associated molecular patterns released after tissue/cellular injury are capable of activating dermal and splenic γδ T cells, leading to increased TLR expression and cytokine, chemokine, and growth factor release [30, 31]. Our data contribute to the published role of *HMGB1* in amplifying inflammation [32–34].

Our study has some limitations. As mentioned above, the ultimate proof of γδ T-cell infiltration in human kidneys early after AKI may be provided only by timed kidney biopsies that were not available because of the design of our observational trial. Furthermore, although the nine patients in each study group showed unambiguous results, the sample size was small. We also point out that the mouse IRI model and the corresponding homogeneous cohort of patients undergoing endovascular aortic surgery may not be generalizable to all patients with AKI.

## Conclusions

Our bedside-to-bench approach is the first demonstration that γδ T cells are involved in the early immune response after renal cell injury brought on by IRI in both mice and humans. These cells traffic to the kidney, and their decrease in blood correlates with a greater level of tubular cell injury evidenced by TIMP2 and IGFBP7. *HMGB1* is released from injured renal cells and may further amplify the immune reaction. These findings support the hypothesis that the immune response is initiated immediately after cellular stress/injury before AKI becomes evident, and the future immunological interventions may rather be triggered by early cellular injury markers than by standard markers of kidney function. The results of our study may pave the way for future investigation of γδ T cells in biomarker-positive patients in larger cohorts of patients. Because human AKI is difficult to prevent in the clinical setting, γδ T cells may represent an early marker of systemic immune activation in early AKI and a possible target for future early interventions.

## Additional files

Additional file 1: **Figure S1. a** Schematic overview of the study. **b** Creatinine change over time, stratified according to the risk score. Patients with elevated biomarkers showed a marked creatinine increase on day 1 compared with their baseline values. Data are mean with SEM. *p* = 0.021, Mann-Whitney U test. Biomarker-negative patients had no relevant creatinine alteration. **c** Relevant baseline parameters of study patients, stratified by risk score immediately after surgery. Data are given as median with IQR. *n.s.* Not significant. *p < 0.05. (JPG 120 kb)

Additional file 2: **Figure S2.** Perioperative changes (postsurgery versus presurgery) of T-cell subsets in human peripheral blood, including γδ T cells, were correlated with biomarker levels ([TIMP-2]•[IGFBP7]). In contrast to Fig. 1c, data are shown as absolute cell numbers. Again, no significant associations were observed between cell number differences and biomarker levels for CD3, CD4, or CD8 T-cell subsets. In the γδ T-cell compartment, however, the extent of cell number differences was significantly correlated with biomarker values (*p* = 0.01). (JPG 90 kb)
