# Supplementary figures and images for: Postoperative cellular stress in the kidney is associated with an early systemic γδ T-cell immune cell response
Source: Crit Care. 2018 Jul 4;22:168. doi: 10.1186/s13054-018-2094-x (PMC6030780; doi:10.1186/s13054-018-2094-x)

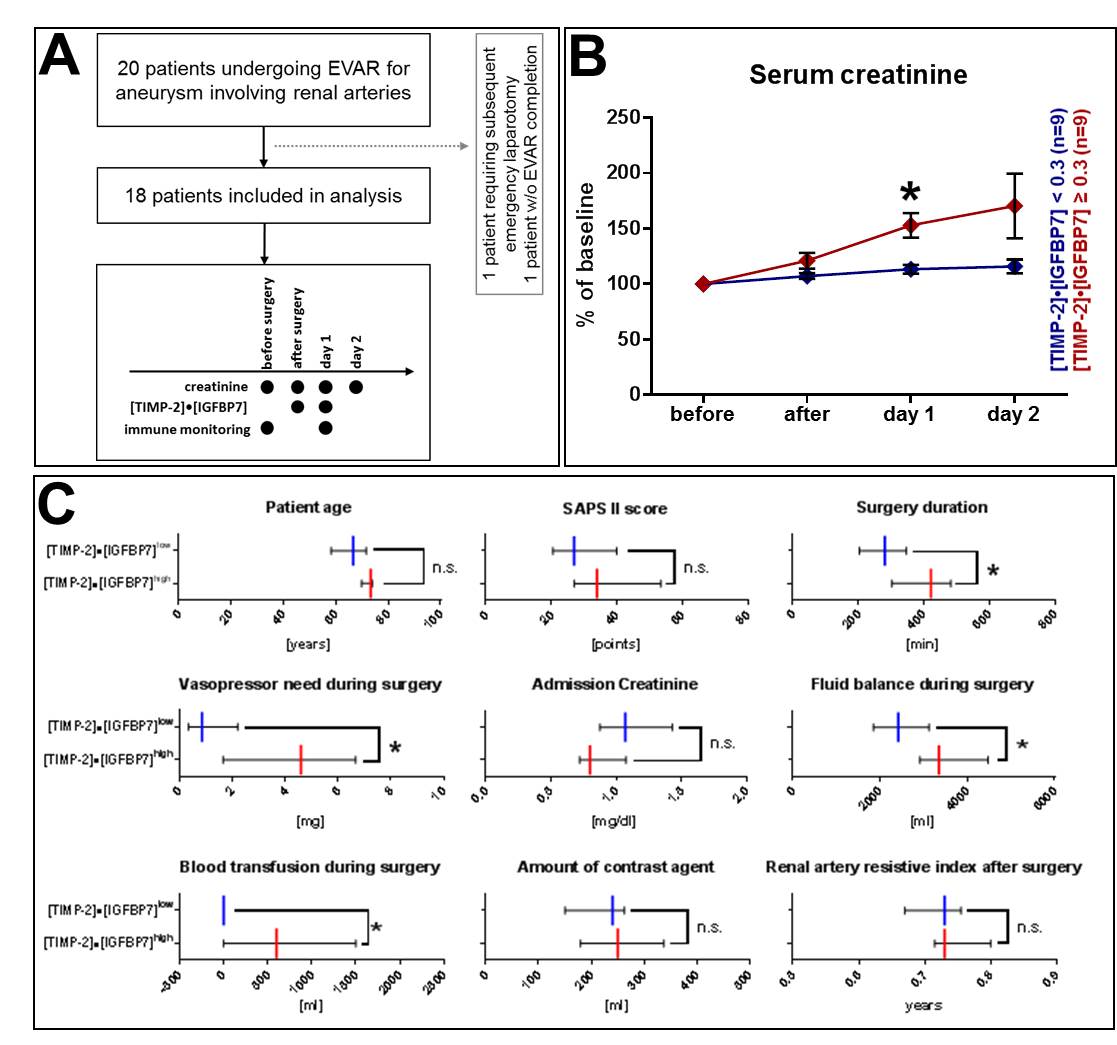

Supplement: Supplementary file 1 — Figure S1. a Schematic overview of the study. b Creatinine change over time, stratified according to the risk score. Patients with elevated biomarkers showed a marked creatinine increase on day 1 compared with their baseline values. Data are mean with SEM. p = 0.021, Mann-Whitney U test. Biomarker-negative patients had no relevant creatinine alteration. c Relevant baseline parameters of study patients, stratified by risk score immediately after surgery. Data are given as median with IQR. n.s. Not significant. *p < 0.05. (JPG 120 kb) [file 13054_2018_2094_MOESM1_ESM.jpg]

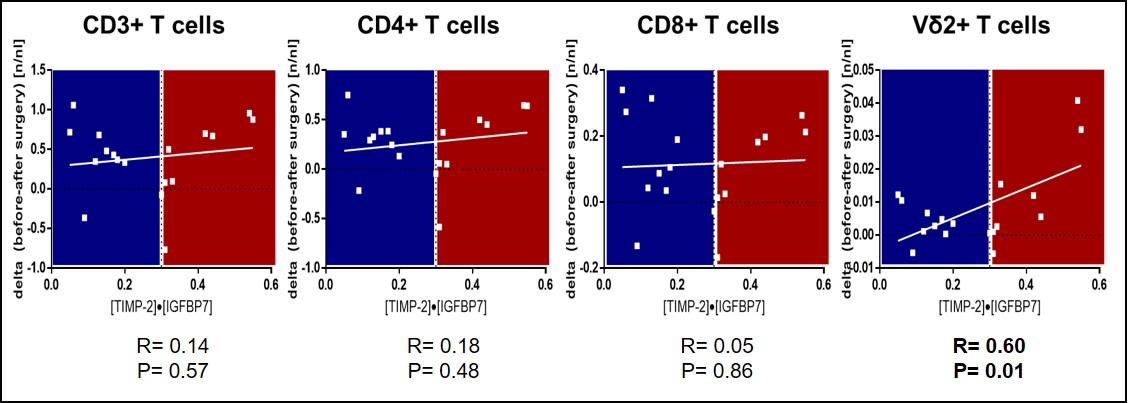

Supplement: Supplementary file 2 — Figure S2. Perioperative changes (postsurgery versus presurgery) of T-cell subsets in human peripheral blood, including γδ T cells, were correlated with biomarker levels ([TIMP-2]•[IGFBP7]). In contrast to Fig. 1c, data are shown as absolute cell numbers. Again, no significant associations were observed between cell number differences and biomarker levels for CD3, CD4, or CD8 T-cell subsets. In the γδ T-cell compartment, however, the extent of cell number differences was significantly correlated with biomarker values (p = 0.01). (JPG 90 kb) [file 13054_2018_2094_MOESM2_ESM.jpg]
